# *Salmonella enterica* serovars co‐exist sporadically and at low abundance in US mid‐Atlantic irrigation ponds

**DOI:** 10.1002/jsfa.70731

**Published:** 2026-05-12

**Authors:** Shirley A Micallef, Mary Theresa Callahan, Nikki W Shariat, Yisrael Katz, Xingchen Liu

**Affiliations:** ^1^ Department of Plant Science and Landscape Architecture College of Agriculture and Natural Resources, University of Maryland College Park MD USA; ^2^ Centre for Food Safety and Security Systems, University of Maryland College Park MD USA; ^3^ Department of Population Health College of Veterinary Medicine, University of Georgia Athens GA USA

**Keywords:** irrigation pond water, CRISPR‐SeroSeq, *Salmonella* quantification, food safety, microbial water quality, agricultural water assessment

## Abstract

**BACKGROUND:**

Spring or rain‐fed ponds are frequent features on US mid‐Atlantic farms. The need to conduct preharvest agricultural water assessments to identify microbial hazards, as required by the US Produce Safety Rule, highlights knowledge gaps pertaining to pond water adequacy for vegetable production. We sought to evaluate *Salmonella enterica* in Maryland farm ponds during the vegetable‐growing season.

**RESULTS:**

*Salmonella* was isolated and semi‐quantified by selective culture and *Escherichia coli* was enumerated on MI agar, from 60 water samples collected from 21 ponds on 11 farms from June to October. Aliquots of pre‐enrichment/enrichment broths that yielded *Salmonella* isolates by selective culture were subjected to serotyping with CRISPR‐SeroSeq. *Salmonella* was undetectable in 11 of the 21 ponds, while 10 ponds from seven of the 11 farms were positive at least once. Farm, but not month of collection, was a significant factor for *Salmonella* detection but not for *Salmonella* estimates. Levels ranged from 0.03–2.9 MPN L^−1^ of water, with only three samples exceeding 1 MPN L^−1^. Eleven serovars were identified, with type and richness differing by month. Individual ponds yielded one to seven serovars. August and October had the highest richness, with *Salmonella* Enteritidis and Infantis dominating. Farm was a factor for *E. coli* counts, which was quantified in all but one sample. No relationship with *Salmonella* levels was discernible.

**CONCLUSION:**

Low levels of *Salmonella* and sporadic detection suggest that pond water could be amenable to treatment and serve as a manageable, safe, sustainable and affordable preharvest agricultural water type. The information can support vegetable farmer decisions to consider ponds as viable water sources. © 2026 The Author(s). *Journal of the Science of Food and Agriculture* published by John Wiley & Sons Ltd on behalf of Society of Chemical Industry.

## INTRODUCTION

Natural or man‐made ponds are an important irrigation water source for fresh produce crops in the Mid‐Atlantic, USA. However, the use of surface water, including pond water, has declined in recent years as farmers have grown uncertain about the microbial quality of pond water for irrigation, preferring groundwater.^1^, ^2^ Data on pond water quality is crucial for safe, continued use of these irrigation sources and for the long‐term conservation of groundwater resources. In a survey of the fresh produce industry community, including respondents from grower, packer, buyer, consultant and other industry associated groups, ‘preharvest water’ was ranked seventh in importance out of 24 food safety priorities. In the same survey, members of a Produce Advisory Committee (PAC) that comprised high‐level managers and regulators of food safety ranked ‘preharvest water’ a close second in importance to ‘adjacent land’^3^ for fresh produce safety.

Data on the microbial quality of pond water in the mid‐Atlantic region has been published but is frequently reported in conglomerate with other surface water types, preventing the ability to evaluate pond water specifically. Studies have identified 70–78% of surface water samples to be positive for *Escherichia coli* with levels around 2–2.5 log CFU in every 100 mL of water.^4^, ^5^ Surface water is typically of lower microbial quality than groundwater from well‐maintained wells, which often have undetectable levels of *Escherichia coli* and *Enterococcus* spp. in 100 mL of water.^4^, ^5^, ^6^ Additionally, *Salmonella* prevalence was reported to be higher in surface waters, including ponds, compared to well water.^7^, ^8^ The microbial quality of ponds, however, being lentic systems recharged from spring water or surface run‐off from a small watershed, stands to be superior to lotic systems such as rivers and creeks, with more widespread watersheds encompassing variable land uses. Microbial reports from the mid‐Atlantic support this premise, with lower levels of generic *Escherichia coli*, *Enterococcus* spp. and *Aeromonas* spp., lower diversity of *Aeromonas* spp. and lower or undetectable presence or levels of *Salmonella enterica* and *Listeria monocytogenes* in ponds compared to rivers.^8^, ^9^, ^10^, ^11^ In a study assessing whether mid‐Atlantic water sources would comply with microbial water standards for food safety, pond water sites met the *Escherichia coli‐*based thresholds that are recognized in US agricultural water regulation.^10^, ^12^

Despite indications that pond water may be a safe water source to use for irrigation of fruit and vegetable crops, grower trepidation in using this water source in the mid‐Atlantic also stems from knowledge that testing for *Escherichia coli* may not be an adequate harbinger for foodborne pathogens of concern, particularly *S. enterica*, which has caused fresh produce‐linked outbreaks in the larger mid‐Atlantic region.^13^, ^14^ Moreover, recent changes to regulations pertaining to irrigation water quality have brought a shift from testing agricultural water for *Escherichia coli* levels to conducting preharvest agricultural water assessments that do not require mandatory *Escherichia coli* testing.^15^ These hazard assessments are now the predominant approach to identify scenarios under which water may be unsafe for use on fresh produce crops. However, data on enteric pathogen dynamics in irrigation ponds, and factors driving these dynamics, are largely lacking, despite being crucial information to conduct preharvest agricultural water assessments to identify hazards that may compromise water quality.

The current state of affairs regarding on‐farm pond water in the mid‐Atlantic region calls for more research to support preharvest agricultural water assessments and decision making in crop management, irrigation and use of this abundant and free water source. In view of this need, our objective was to contribute specific data on the microbial quality of pond water in Maryland, USA. We therefore collected samples from 21 on‐farm ponds in Maryland during the vegetable crop growing season to quantify and characterize *S. enterica* populations in water samples, and to evaluate the relationship of *Salmonella* populations with levels of generic *Escherichia coli*.

## MATERIALS AND METHODS

### Study sites

Eleven small farms in Maryland were recruited for the study, allowing for the sampling of 21 irrigation ponds at least once, and up to four times between June and October 2019, generating a total of 60 samples. A questionnaire was administered at the first site visit to gather information about each pond and farm. Since the sites included commercial farms, the study was reviewed by the University of Maryland College Park Institutional Review Board (IRB) (project number 1411872) and was approved as a minimal risk project to farm owners.

### Water sampling

For enumeration of generic *Escherichia coli*, bulk water samples (1 L) were collected in sterile bottles. For semi‐quantitation of *Salmonella*, 10 L, 1 L and 0.1 L were filtered on site in triplicate through Modified Moore swabs made of cheesecloth, as previously described.^16^, ^17^, ^18^ Water and swab samples were transported back to the laboratory in coolers containing ice, and sample processing was initiated within 4 h of collection.

### Bacterial quantitation

Generic *Escherichia coli* was enumerated by standard membrane filtration according to the Environmental Protection Agency (EPA) Method 1604 and as previously described.^19^ For *Salmonella* quantification, 100 mL of Lactose broth (LB, Criterion, Hardy Diagnostics, Santa Maria, CA, USA) was added to each Modified Moore swab in sterile Whirl‐pak bags, hand massaged for 2 min and placed at 37 °C for 24 h. Then, 1 mL of this pre‐enrichment was transferred to 9 mL tetrathionate (TT) broth (Becton, Dickinson and Co. (BD), Franklin Lakes, NJ, USA) with iodine.^8^ Tubes were vortexed and placed at 42 °C for 24 h, then 10 μL of each enrichment were streaked onto XLT4 agar (BD). Plates were incubated at 37 °C for 24 h. Estimates of most probable number per litre of water (MPN L^−1^) were calculated based on presence/absence of *S. enterica* in three replicates of three water volumes (0.1 L, 1 L and 10 L), using the EPA MPN calculator.^20^ The limit of detection was 0.03 MPN L^−1^. Negative samples were assigned a concentration of half the limit of detection, rounded to 0.02 MPN L^−1^.

### Serotyping with CRISPR‐SeroSeq

For all samples that were positive for *Salmonella* by selective culture, 1.5 mL aliquots of the LB and TT broth suspensions were centrifuged for 2 min at 13225 × *g* to retrieve the pellet to be used in DNA extraction using the Qiagen DNeasy microbial kit (Qiagen, Hilden, Germany). A total of 2 μL was used as a template for CRISPR‐SeroSeq. Libraries were generated using a two‐step polymerase chain reaction (PCR).^21^ Amplicons were purified using the AMPure bead system (Beckman Coulter, Brea, CA, USA) and samples were pooled in approximate equimolar ratios based on gel electrophoresis of 5 μL of PCR product. Libraries were sequenced on an Illumina NextSeq along with three controls (a negative for each PCR (water controls), and a positive control containing *Salmonella* Enteritidis genomic DNA as a template). Sequence reads were parsed through a script that uses BLAST to match sequence reads to a database of CRISPR spacer sequences from over 130 serovars, before writing the output directly to Excel and calling serovars as previously described.^21^ CRISPR‐SeroSeq was performed on 37 samples (19 TT and 18 LB). All but two TT libraries failed to generate enough sequence depth while only six LB libraries generated usable sequence data. We suspect this is due to the non‐selective enrichment of LB and subsequent presence of other microbes that may limit the sensitivity of the CRISPR‐SeroSeq PCR.

### Statistical analysis

Chi‐squared (*χ* ^2^) analysis was conducted to test the null hypothesis that presence or serovar of *Salmonella* in water samples was not dependent on month of sample collection or farm. Levels of log transformed *Escherichia coli* counts and *Salmonella* MPN estimates in water samples were fit to mixed models where fixed effects were month, farm and pond, and random effects accounted for the repeated structure of pond sampling. Analyses were conducted in JMP® Pro version 19.0.1 and *α* was set at 0.05. These two datasets were also assessed using Pearson correlation analysis. Using Primer 6 version 6.1.15, ANOSIM was performed on a similarity matrix, constructed from the *Salmonella* positive dataset with the Bray–Curtis coefficient, to determine whether month was a dependent factor for the *Salmonella* serovar profiles obtained.

## RESULTS

### Irrigation pond characteristics

**Figure 1:**
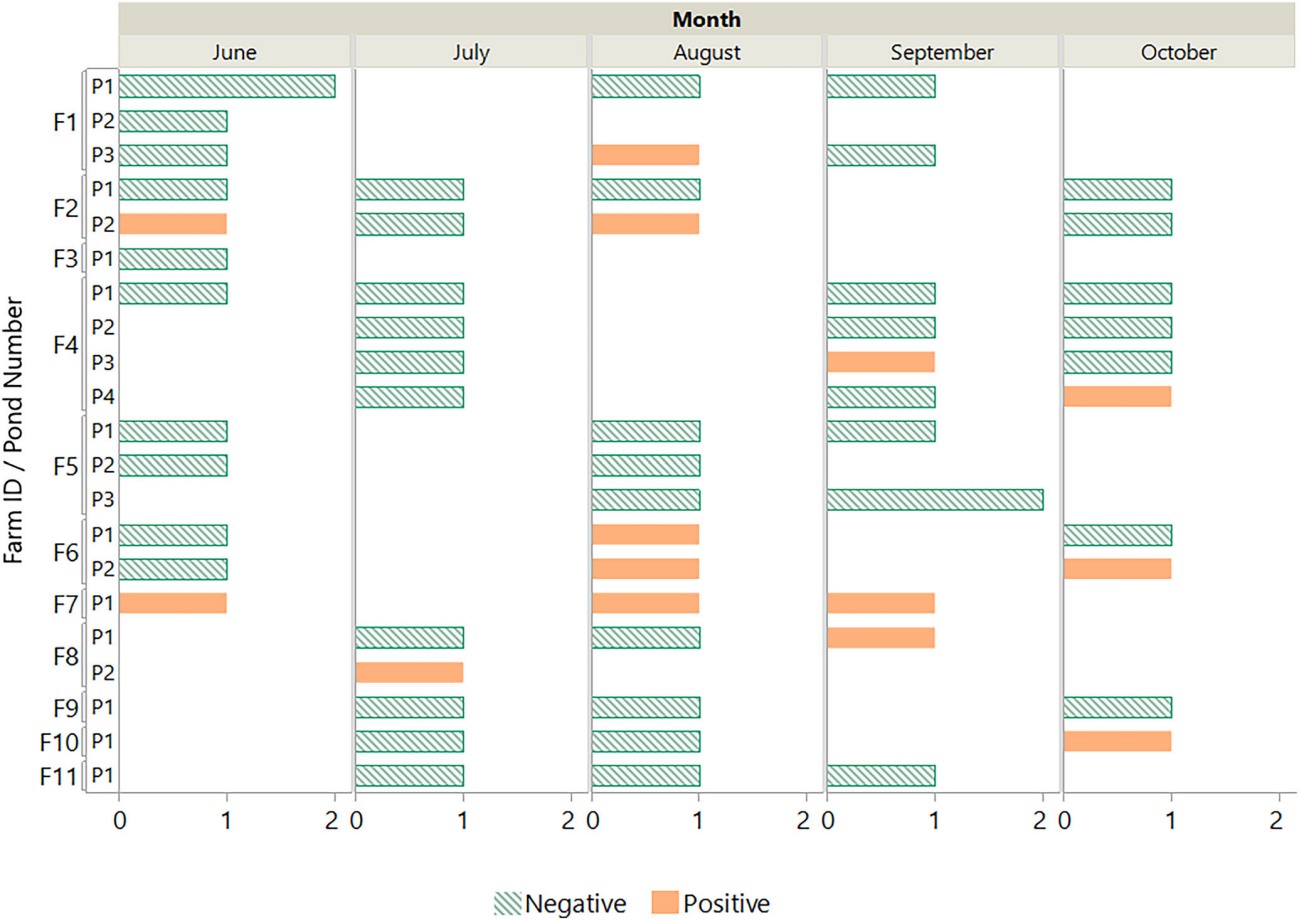
Frequency of *Salmonella enterica* detection by selective culture in 21 ponds on 11 farms in Maryland, sampled between June and October. F and P in pond codes refer to farm and pond number, respectively.

Twenty‐one ponds on 11 farms (F1–F11) were sampled between one to four times – four ponds were sampled four times, 13 were sampled three times, one pond was sampled twice and three were sampled only once (Table 1 and Fig. 1). Out of these 21 ponds, all but one were situated on farm property, with one being on shared property. One pond was reported to be recharged primarily from runoff, 11 were spring fed and nine were reported to be recharged by a combination of spring water and runoff. The banks of the ponds were vegetated entirely by grassland (14 ponds) or woods (five ponds) or a combination of trees and grassy areas (two ponds). Eight ponds had very steep banks while the rest had a bank that sloped between 5° and 40°. Proximal adjacent land use at each farm included a mix of agricultural land (15/21; 71%), trees (11/21; 52%) or woods (7/21; 33%), grassland (3/21; 14%) or residential areas (2/21; 9%). At the time of sampling, 12 ponds were actively being used for irrigation of fresh produce (10/21 ponds), berries (2/21) or fruit trees (1/21). All used drip irrigation, with two farms reporting using overhead irrigation to germinate seedlings. Growers reported diverse wildlife accessing the pond, including fish (90%), amphibians, especially frogs and toads (43%), birds such as geese (76%), reptiles – especially turtles (14%) and mammals – especially deer (29%). Two farms reported poultry (F4_P1) and cattle (F4_P2) rearing activities occurring in the vicinity of their farms.

**Table 1:** Ponds sampled in this study and their characteristics

Farm	Pond	Pond recharge	Used for irrigation	Bank vegetation	Adjacent land use
F1	P1	Spring fed	No	Grasses	Trees, agricultural land
	P2	Spring fed	No	Grasses	Trees, agricultural land
	P3	Spring fed	fresh produce	Grasses	Agricultural land
F2	P1	Spring fed	Berries	Grasses	Agricultural land
	P2	Spring fed	Fruit trees, berries	Grasses	Trees, agricultural land
F3	P1	Spring fed, runoff	Fresh produce	Grasses	Agricultural land, residential area
F4	P1	Spring fed, runoff	Fresh produce	Grasses	Trees, agricultural land
	P2	Spring fed, runoff	Fresh produce	Grasses	Trees, agricultural land
	P3	Spring fed, runoff	Fresh produce	Grasses	Trees, agricultural land
	P4	Spring fed, runoff	Root vegetables, herbs, leafy greens, produce	Grasses	Trees, agricultural land
F5	P1	Spring fed, runoff	Fresh produce	Woods	Wooded area
	P2	Spring fed, runoff	Fresh produce	Woods	Wooded area
	P3	Spring fed, runoff	No	Woods	Wooded area
F6	P1	Spring fed	No	Grasses	Trees, grassy land
	P2	Spring fed	Fresh produce	Grasses	Trees, grassy land
F7	P1	Spring fed	No	Grasses	Agricultural land, wooded area
F8	P1	Spring fed	No	Woods	Agricultural land, trees, residential area
	P2	Runoff	Fresh produce	Woods	Agricultural land, wooded area
F9	P1	Spring fed	No	Grasses	Trees, grassy land
F10	P1	Spring fed	No	Grasses, trees	Agricultural land, wooded area
F11	P1	Spring fed, runoff	No	Grasses, woods	Agricultural land, wooded area

### *Salmonella* occurrence in pond water

The sampling scheme described earlier generated a total of 60 water samples. *Salmonella* was never detected in 11 out of the 21 ponds, sampled for a total of 31 times (Fig. 1). The other ten ponds (47.6%), located on seven out of 11 farms (63.6%), were found to be positive for *Salmonella* at least once (14 *Salmonella‐*positive samples out of 29 sampling events for those ponds). Overall, therefore, 14 out of 60 (23.3%) water samples yielded *Salmonella* isolates. Except for pond F8_P2 sampled only once, the *Salmonella*‐positive ponds were sampled at least three times, with one pond (F2_P2) sampled four times. *Salmonella*‐positive status for any given pond was sporadic. *Salmonella* presence was detected once in seven ponds (six of which were sampled three times), twice in two ponds and three times (100%) in pond F7_P1 (Fig. 1). For any given month, farms with multiple ponds had a mix of *Salmonella‐*positive and negative ponds (F1, F2, F4, F8), *Salmonella‐*positive ponds only (F6), or *Salmonella‐*negative ponds only (F1, F2, F4, F5, F6) (Fig. 1). Farm was a factor for whether *Salmonella* was detected (*χ* ^2^ (10, *N* = 60) = 21.32, *P* < 0.05). *Salmonella* was detected in every month sampled at a rate of 15%, 9%, 36%, 25% and 30% from June to October, respectively. Despite the slight increase in *Salmonella‐*positive detection in August–October, month of sample collection was not a significant factor for *Salmonella* occurrence (*χ* ^2^ (4, *N* = 60) = 3.37, *P* > 0.05).

### *Salmonella* serovar diversity

**Figure 2:**
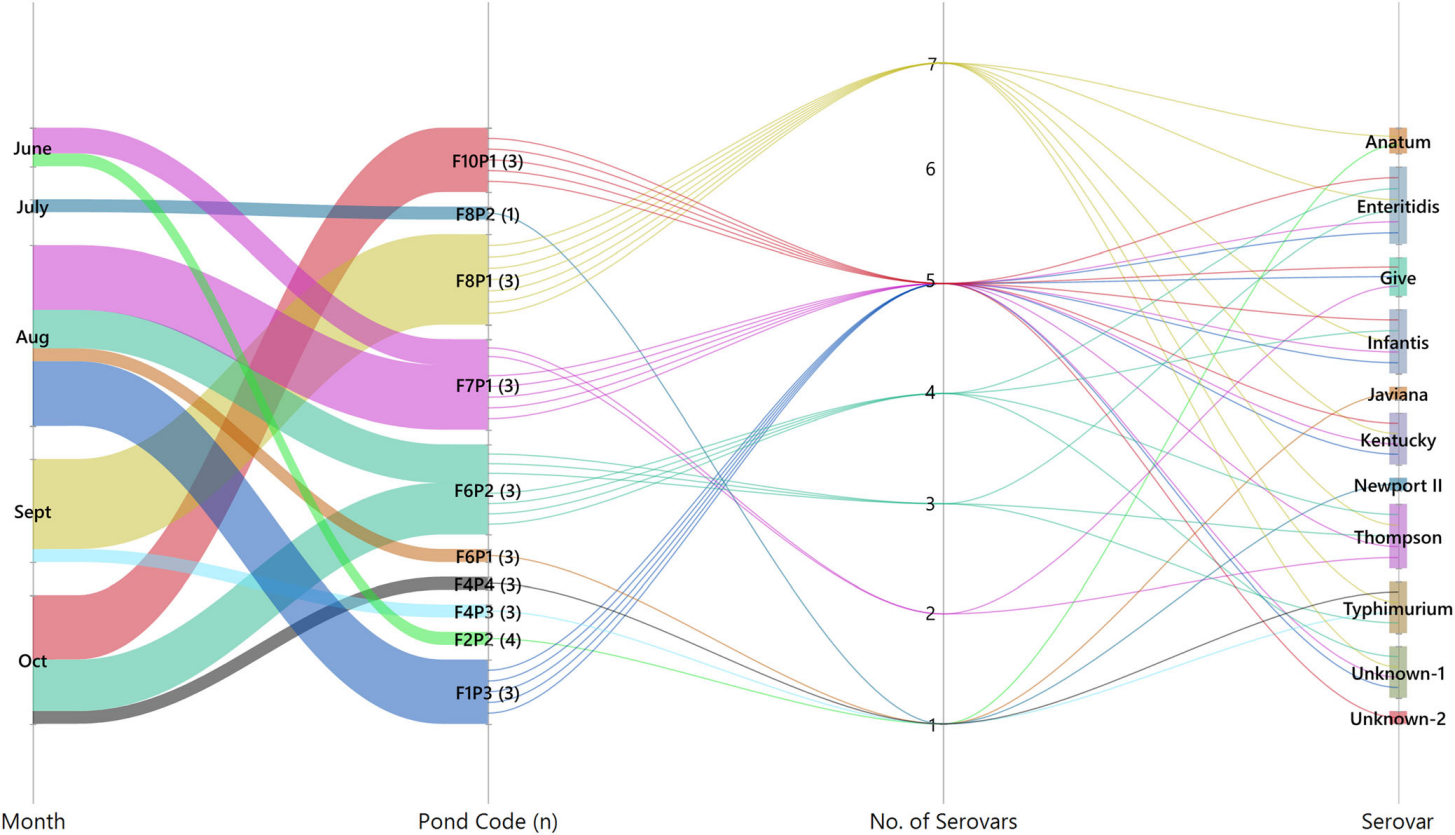
Parallel plot displaying ponds that yielded *Salmonella* isolates confirmed by CRISPR‐SeroSeq, grouped by month of sampling and differentiated by *Salmonella* serovar. The plot is colour‐coded by pond. The number (*n*) in parenthesis next to the pond code indicates the total number of times that pond was sampled, with *Salmonella‐*positivity shown in Fig. 1.

Eleven distinct *Salmonella* serovars were identified, with nine being classified as a known serovar and two remaining unclassified (Fig. 2). *Salmonella* Enteritidis and Infantis were the most frequently detected serovars, occurring together in five different ponds in August, September and October (Fig. 2). *Salmonella* Kentucky, Typhimurium and an untypeable serovar (unknown‐1) also occurred in these months and were detected in four different ponds. *Salmonella* Javiana was also recovered from one pond in August, and a second untypeable serovar (unknown‐2) was recovered from one pond in October. August and October yielded the most serovar richness, with 11 and 10 serovars, respectively. Only one serovar (*Salmonella* Newport II) was detected in July, while three serovars, *Salmonella* Anatum, Give and Thompson, were identified in June. The effect of month on serovar detected approached significance (*χ* ^2^ (40, *N* = 36) = 54.64, *P* = 0.06).

As reported earlier, most ponds were found to be positive for *Salmonella* only once out of multiple sampling months. However, most samples were characterized by high serovar diversity. The pond with the highest serovar richness was F8_P1, which was sampled three times but yielded seven serovars, all in the September sampling (Fig. 2). Likewise, F1_P3 and F10_P1, sampled three times, yielded five serovars only in the August and October sampling, respectively. Two ponds yielded different *Salmonella* serovar assemblages on different sampling occasions, specifically F6_P2 and F7_P1. Despite this, there were some similarities. Serovar Thompson was found in two of three F7_P1 samplings (June and August) and serovars Enteritidis and Thompson were found in both the *Salmonella* positive F6_P2 samplings (August and October). Sequencing of samples from pond F2_P2 only gave *Salmonella* positive results for the June sampling, while the August sample was negative. The remaining ponds yielded one serovar during one sampling event only (Fig. 2). Interestingly, these serovar identifications came from TT broth‐extracted DNA, which may signal a selective bias for that medium. *Salmonella* Javiana, Newport II, Typhimurium and the untypeable serovar ‘unknown‐2’ were detected in these single‐serovar samples. *Salmonella* Enteritidis and Infantis always co‐occurred, while *Salmonella* Give, Kentucky and the untypeable serovar ‘unknown‐1’ consistently occurred with these former two serovars, but not vice versa. Using ANOSIM, we found no evidence that the *Salmonella* serovar groupings obtained in each sample were related to month of collection (*R* = 0.012, *P* > 0.1).

### *Salmonella* semi‐quantification and relationship with *Escherichia coli* concentrations

**Figure 3:**
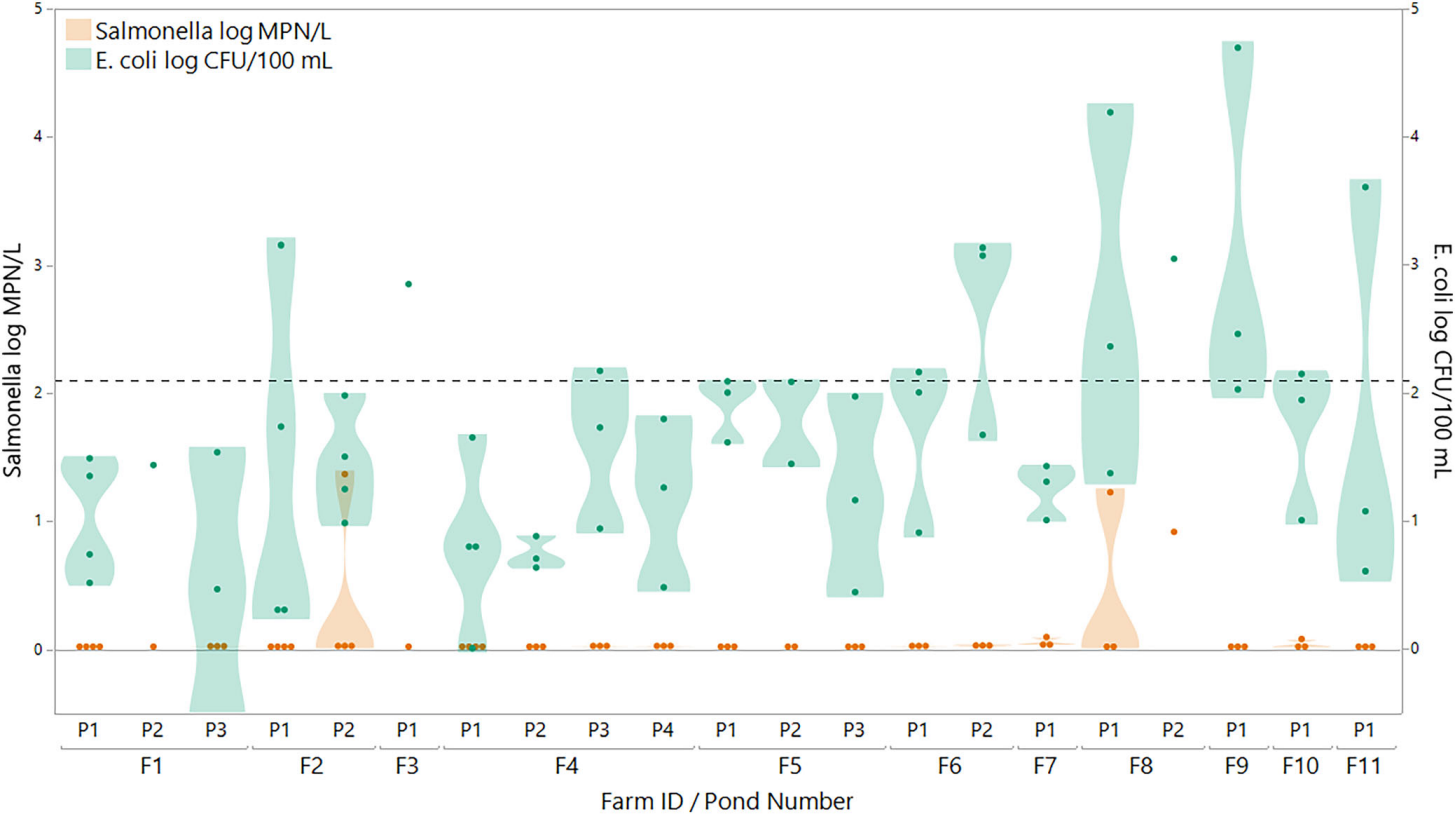
*Salmonella* MPN estimates (represented in log (MPN + 1), left *y*‐axis) and *Escherichia coli* counts (represented in log CFU in 100 mL, right *y*‐axis) for each pond, ordered by farm. The dashed line indicates the threshold for *Escherichia coli* geometric mean counts of 126 CFU in 100 mL (2.1 log CFU in 100 mL) adopted by some US states.

**Figure 4:**
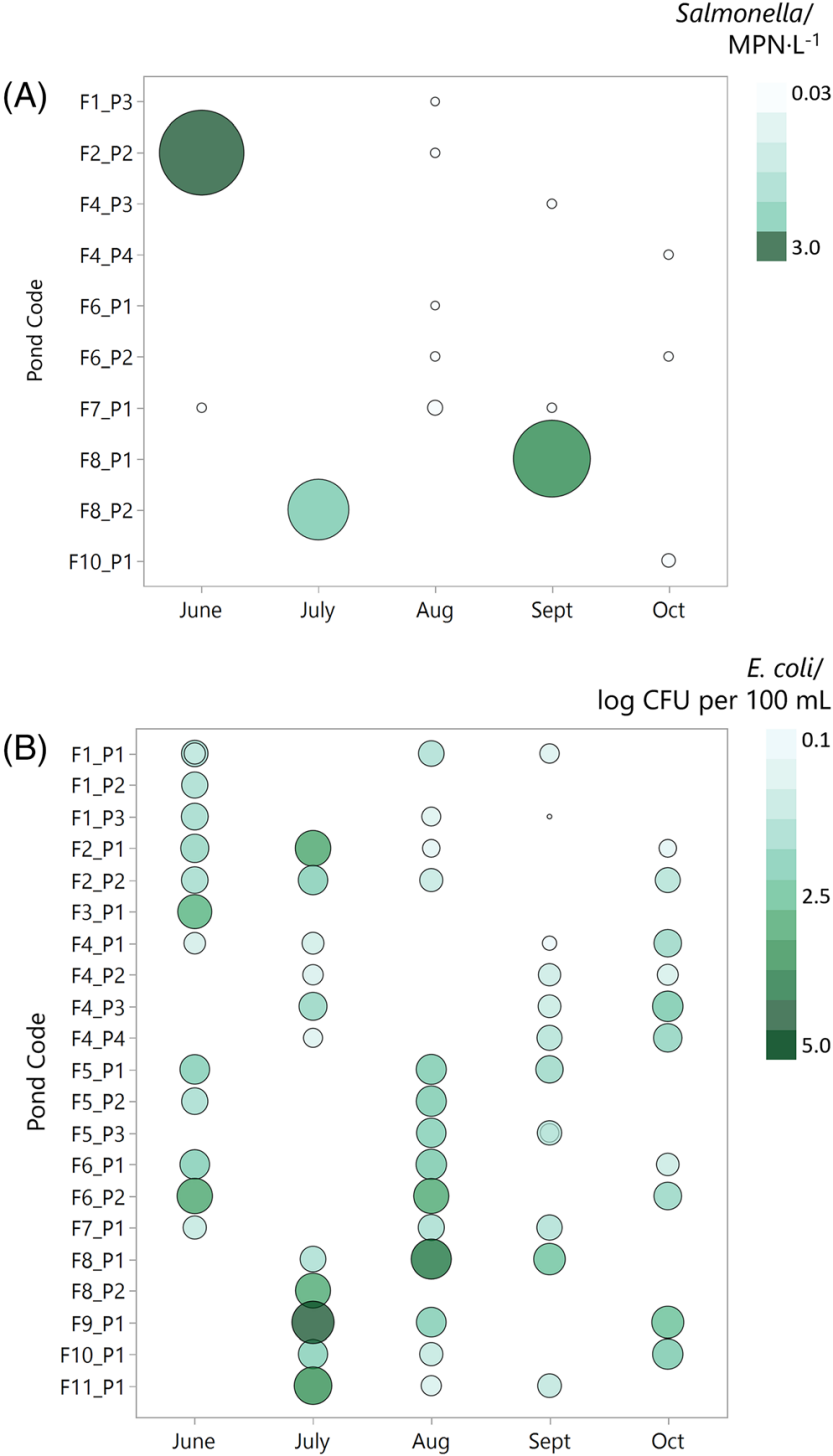
Bubble plots of (A) *Salmonella* and (B) *Escherichia coli* levels in *Salmonella*‐positive samples, distributed by month.

The estimated MPN for *Salmonella* ranged from 0.03–2.92 MPN L^−1^ of water (mean of 0.14 ± 0.07 MPN L^−1^; Table 2 and Fig. 3). The distribution of *Salmonella* population estimates was skewed to the lower end of this range (*n* = 9 at < 0.04 MPN L^−1^) and only three samples exceeded 1.0 MPN L^−1^ – all from different ponds, including two ponds from the same farm (F8) but from different months (Fig. 4(A)). The estimated *Salmonella* population levels were not dependent on month of collection or pond, though farm may have had a weak effect that was not supported statistically (*P* = 0.1), driven by differences between F8 *versus* F1 and F5 (both *P* = 0.06). *Escherichia coli* was detected in all but one sample from F4_P1 collected in September. The highest *Escherichia coli* concentrations measured was 4.7 log CFU in 100 mL of sample and the mean was 1.6 ± 0.13 log CFU in 100 mL. Higher *Escherichia coli* levels were measured in July (2.1 ± 0.4 log CFU in each 100 mL) than September (0.9 ± 0.2 log CFU in 100 mL; *P* < 0.05). *Escherichia coli* counts were dependent on farm (*F*‐ratio = 2.7, *P* ≤ 0.01), whereby farm differences were revealed, notably farm F9 compared to F4 (*P* ≤ 0.5) and F1 (*P* = 0.07; Fig. 3). Three out of 18 ponds with two or more samples for which a mean could be computed exceeded the *Escherichia coli* geometric mean (GM) standard of 126 CFU in 100 mL (2.1 log CFU in 100 mL) previously specified in the Food Safety Modernization Act (FSMA), Produce Safety Rule^15^ and still applied in Maryland Department of Agriculture (MDA) Good Agricultural Practices (GAPs) audits.^22^ Two ponds with *n* = 1 gave a measurement that exceeded the GM standard (Fig. 3). Of these, F8_P1 and F8_P2 were also positive for *Salmonella*. There was only a 0.16 log difference in *Escherichia coli* levels between *Salmonella* positive (mean of 1.70 ± 0.21 log CFU in 100 mL; *n* = 14) and negative (mean of 1.54 ± 0.16 log CFU in 100 mL; *n* = 46) samples. No correlation was discernable between *Salmonella* and *Escherichia coli* levels, even when data from *Salmonella* negative samples were excluded from the correlation analysis.

**Table 2:** *Salmonella* estimates by farm

Farm code	*n*	MPN L^−1^	SE
F1	8	0.02	0.001
F2	8	0.38	0.36
F3	1	nd	—
F4	13	0.02	0.002
F5	8	nd	
F6	6	0.03	0.003
F7	3	0.06	0.02
F8	4	0.98	0.59
F9	3	nd	—
F10	3	0.04	0.02
F11	3	nd	—

*Note*: Means were calculated only for farms that had at least one positive sample. *Salmonella* negative samples were assigned a concentration of 0.02 MPN L^−1^. SE denotes standard error; *n* denotes number of samples; nd denotes that *Salmonella* was never detected at that farm.

## DISCUSSION

This study evaluated the presence, diversity and level of *S. enterica* in irrigation ponds on small scale Maryland farms located in the mid‐Atlantic region of the United States. A little less than half of the ponds were found to harbour *Salmonella* at least once, but occurrence in most ponds was sporadic during the crop growing season and levels detected were of very low concentration. At the same time, several ponds were found to support multiple *Salmonella* serovars, especially in the later months of August to October. Moreover, when the same pond was positive more than once, the majority of identified serovars differed by month for that pond. Taken together, these findings could indicate either multiple reintroduction events, or persistent populations that exist below the detection limit that ebb and flow in response to ecosystem dynamics. With farm being a factor for *Salmonella* detection and *Escherichia coli* levels, risk determinants may exist at specific farms that either increase the likelihood of sporadic pathogen or faecal indicator introduction events or provide favourable conditions that allow for undetectable *Salmonella* to regrow.

These findings are relevant to the Agricultural Water Assessments that are now required to be conducted at the start of each growing season to comply with current preharvest agricultural water regulation in the United States.^15^ These assessments are intended to help identify conditions that could point to immediate or predictable hazards. Several factors need to be considered, including type of water source, water distribution and delivery, adjacent land use and potential activities outside the farm's control. Data on bacterial/pathogen population dynamics, as well as genetic variability, in ponds can help better assess risks associated with an event that may have introduced microbial pathogens. Previous research has reported more *Enterococcus faecalis* phylogenetic similarity within than among ponds that are spatially separated across the mid‐Atlantic region.^6^ Additionally, the variance in levels of faecal indicator bacteria (FIB) retrieved from closely situated ponds was more influenced by pond conditions, as opposed to spatial influences governing the variance of FIB in ponds that were located farther apart.^23^ Data specific to *Salmonella* from ponds investigated in this study also identified farm as a significant factor in *Salmonella* detection. These data add to our understanding of the microbial ecology of different bacterial groups and may prove relevant to agricultural water assessments on farms.

*Salmonella* is well known for its ability to persist in non‐host environments, especially in surface water bodies where it is frequently detected.^8^, ^9^, ^17^, ^24^, ^25^, ^26^ With increasing frequency of dryness or droughts in the mid‐Atlantic region (National Oceanic and Atmospheric Administration (NOAA) climate report 2023), irrigation during the crop growing season is imperative to maintain crop production. The frequency of *Salmonella* foodborne disease via contaminated fresh fruit and vegetable consumption has been linked to preharvest water,^14^, ^24^, ^27^ such that pathogen presence in ponds raises food safety concern. *Salmonella* abundance measured in this study was comparable or lower than previously reported for this region,^9^, ^28^ while serovar diversity was comparable or higher.^8^, ^28^ With improved methods for water sampling that filter larger volumes of water, reports of *Salmonella* detection have increased,^9^ shifting our understanding of *Salmonella*'s frequent occurrence in surface waters. Our finding of lower incidence may also be explained by our choice of microbiological media; the use of modified semi‐solid Rappaport Vassiliadis Agar aided the recovery of *Salmonella* from surface water in other studies.^29^, ^30^ Nonetheless, the CRISPR‐SeroSeq approach we employed may have been limited by the challenges of isolating low abundance *Salmonella* from complex water samples since several LB pre‐enrichment suspensions did not yield usable DNA libraries. Such limitations have been noted before in constructing libraries from pre‐enrichments in Buffered Peptone Water.^31^

Nevertheless, this study supports the notion that *Salmonella* is prevalent in the mid‐Atlantic surface water agro‐environment, albeit at levels that may not pose a risk when water is not coming in direct contact with the crop on a repeated basis. What constitutes an acceptable level for irrigation water, however, has not been established. More research is needed to determine whether low *Salmonella* levels in water, for instance below 0.04 MPN L^−1^ as was most frequently estimated in our study, might lead to crop colonization. Moreover, the observational nature of this study calls for more in‐depth research that includes quantitative risk assessments for various farm scenarios, as well as research that carefully evaluates the efficacy of any pond water treatments.

The month to month variability we encountered in mid‐Atlantic ponds in this study was also noted for ponds in the south‐eastern United States.^32^, ^33^, ^34^ A seasonal effect could be expected, since *Salmonella* populations could be knocked down during the cold mid‐Atlantic winters, although a previous study only found this to be the case in one out of two ponds tested year round.^35^ However, the sporadic recovery and diversity of *Salmonella*, when detectable in ponds, was higher than that reported in several other studies^8^, ^32^, ^33^ and indicates that the system is variable. The high diversity, low abundance scenario could suggest that competition among *Salmonella* serovars, and likely several other bacterial taxa, play a role in population interactions. The mechanism controlling *Salmonella* may be competition, and management practices that are not detrimental to microbial diversity in ponds, that effectively keep *Salmonella* spp. in check, could be an important safeguard. Adopting drip irrigation practices as opposed to overhead irrigation and limiting the use of surface water later in the season, are also ways that could mitigate contamination risk. The use of adding sanitizer to irrigation water before application to crops is an intervention that has gained acceptance, but the associated costs can be a hurdle for small scale farmers, such that alternative ways to ensure safety while practicing water sustainability are still in demand.

The usefulness of *Escherichia coli* as a predictor for *Salmonella* in surface waters has long been questioned due to competing reports on correlations, or lack thereof, between these two taxa.^26^, ^28^ In our study, we found little evidence to support this relationship. This could be attributed to the sporadic detection of *Salmonella*. The low number of *Salmonella* positive samples overall limited the statistical power of the analysis. However, no association with *Escherichia coli* was found even when only *Salmonella* positive samples were included in the analysis, although the smaller sample size would have undermined the robustness of the analysis. A meta‐analysis that evaluates data from multiple independent sources may be an appropriate approach to more deeply interrogate this relationship. Indeed, however, the large number of ponds lacking detectable *Salmonella* in this study makes the best case for not overlooking this water source for irrigation of crops, especially when crop contact is excluded, as in fruit trees and staked vegetables. Additionally, the low level of *Salmonella* present in pond water might be successfully eliminated with appropriate sanitizer treatment of the preharvest agricultural pond water, though more research is needed to confirm this.

## CONCLUSION

Our study provides strong evidence for the natural occurrence of *S. enterica* in irrigation ponds located in the mid‐Atlantic region of the United States. Serovar diversity shifted in the short‐term, revealing a variable *Salmonella* community. However, despite these fluctuations, *Salmonella* presence was sporadic and abundance was low, maintaining the possibility that the hazard can be managed, although the limited number of *Salmonella* positive samples and observational nature of this cannot be overlooked. Use of pond water is an economical and sustainable practice that relieves pressure from groundwater resources and reduces the reliance on costly municipal water. While the naturally low levels of *Salmonella* should not preclude the use of pond water for low‐risk practices, such as irrigation methods that avoid crop contact, the relatively frequent presence of *Salmonella* in ponds and other surface waters warrants further research that continues to explore the question of safe *Salmonella* thresholds for various agricultural practices.

## Data Availability

The data that support the findings of this study are available from the corresponding author upon reasonable request.
